# Association of dietary fat composition with kidney function decline in patients with type 2 diabetes

**DOI:** 10.1186/1758-5996-7-S1-A36

**Published:** 2015-11-11

**Authors:** Ana Luiza Teixeira dos Santos, Camila Kümmel Duarte, Maira Zoldan, Manoella Freitas Santos, Lorenzo Catucci Boza, Jorge Luiz Gross, Mirela Jobim de Azevedo, Themis Zelmanovitz

**Affiliations:** 1 HCPA, Porto Alegre, Brazil

## Background

Chronic kidney disease is a major microvascular complication of Diabetes Mellitus (DM) and may affect about one third of the patients. Based on the evidence that a proportion of patients may have reduced glomerular filtration rate (GFR) without increased albuminuria, it is recommended to evaluate both parameters. Furthermore, both of them are considered prognostic factors of renal and cardiovascular mortality. Hence, the evaluation of the possible association between dietary fat content and renal dysfunction assessed by GFR is also relevant.

## Objective

To evaluate the association between the dietary fat composition and the decline of GFR in type 2 diabetes patients.

## Materials and methods

In this prospective cohort study the usual diet of patients was assessed by a 3-day weighed diet record (WDR). Compliance with the WDR technique was assessed by comparing protein intake estimated from 3-day WDR and 24-h urinary nitrogen output. GFR was estimated by using the CKD-EPI equation. After a minimum follow up of one year, a new clinical and laboratory evaluation assessment was performed and eGFR was calculated again.

## Results

A total of 368 patients were evaluated (177 [48.1%] male, mean age 60.6±9.7 yrs., duration of diabetes 12.4±8.1 yrs., body mass index 28.5±4.3 kg/m^2^) with an average follow-up time of 6±3 yrs. In baseline, 30% of patients presented Diabetic Kidney Disease. The median decline of eGFR per year was 3 (-51 – 47) ml/min/m2. In multiple linear regression models, adjusting for age, gender, systolic blood pressure and 24-hour UAE, the eGFR decline per year was positively associated with the dietary trans fatty acid intake (% energy) (R2=0.074, β - Standardized Coefficients=0.106, P=0.049). When the patients was separated according to gender, this association remained significant, but only in women (R2=0.112, β -Standardized Coefficients=0.183, P=0.014). No association was observed between other dietary fatty acids and eGFR decline.

## Conclusion

In patients with type 2 diabetes, the decline of the glomerular filtration rate seems to be associated with the dietary consumption of trans fatty acid, especially in females.

